# The Role of Location of Tumor in the Prognosis of the Pancreatic Cancer

**DOI:** 10.3390/cancers12082036

**Published:** 2020-07-24

**Authors:** Mirang Lee, Wooil Kwon, Hongbeom Kim, Yoonhyeong Byun, Youngmin Han, Jae Seung Kang, Yoo Jin Choi, Jin-Young Jang

**Affiliations:** Department of surgery, Seoul National University Hospital, Seoul 03080, Korea; rang5026@snu.ac.kr (M.L.); willdoc@snu.ac.kr (W.K.); surgeonkhb@snu.ac.kr (H.K.); yoonhyeong@snu.ac.kr (Y.B.); views@snu.ac.kr (Y.H.); 74398@snuh.org (J.S.K.); 74401@snuh.org (Y.J.C.)

**Keywords:** pancreatic neoplasm/analysis, pancreatic neoplasm/surgery, tumor location, survival, clinical staging

## Abstract

Identification of prognostic factors is important to improve treatment outcomes in pancreatic cancer. This study aimed to investigate the effect of the location of pancreatic cancer on survival and to determine whether it was a significant prognostic factor. Altogether, 2483 patients diagnosed with pancreatic cancer were examined. Comparative analysis of clinicopathologic characteristics, survival analysis, and multivariate analysis were performed. Cancers of the pancreatic head or the uncinate process were present in 49.5% of patients. The head/uncinate cancers had more clinical T1/T2 tumors (59.4% vs. 35.5%, *p* < 0.001) and a significantly higher 5-year survival rate (8.9% vs. 7.3%, *p* < 0.001) than the body/tail cancers. The 5-year survival rate in patients with head/uncinate cancers was significantly lower in the resectable (*p* = 0.014) and the locally advanced groups (*p* = 0.007). In patients who underwent resection with curative intent, the 5-year survival rate was lower in the head/uncinate group (*p* = 0.046). The overall outcome of the head/uncinate cancers was better than the body/tail cancers, due to the high proportion of resectable cases. In patients who underwent curative resection, the head/uncinate cancers had a higher number of T1/T2 tumors, but worse outcomes. In the multivariate analysis, tumor location was not an independent prognostic factor for pancreatic cancer.

## 1. Introduction

Pancreatic cancer is one of the leading causes of cancer-related mortality in developed countries and one of the most lethal malignant neoplasms worldwide [1]. Its prognosis might be poor, and accurate prediction of the prognosis is important for patients as well as clinicians in the management of pancreatic cancer.

Surgical approach to pancreatic cancer and its prognosis greatly differ according to the tumor location [2,3,4]. Some authors have argued that pancreatic body and tail cancers have a worse prognosis due to delayed diagnosis. Others have reported that according to the tumor stage at diagnosis, pancreatic body and tail cancers showed superior survival than pancreatic head cancers, in localized and resectable tumors. Despite these differences, tumor location was never taken into consideration in any edition of the American Joint Committee on Cancer (AJCC) staging system, since the first edition in 1978. Thus, the effect of location on pancreatic cancer needs to be highlighted.

Several issues related to tumor location need to be scrutinized in depth. One of them is to clarify whether tumor location affects the prognosis of pancreatic cancer and if it does, the manner in which it affects the prognosis. Furthermore, it should be examined whether tumor location affects the prognosis to such an extent that it should be reflected in the staging system of pancreatic cancer. With these questions in mind, the present study aimed to compare the survival outcomes and clinicopathological features of pancreatic cancer, according to its location.

## 2. Results

### 2.1. Patient Demographics and Survival Outcomes

Altogether, 2483 patients were identified. Among these, 1228 patients (49.5%) had tumors in the pancreatic head or the uncinate process (PHU group) and 1255 patients (50.5%) had tumors in the pancreatic body or the tail (PBT group). Demographics and clinicopathological features are summarized in Table 1. The mean age was comparable between the PHU and the PBT groups (64.3 years and 64.0 years, respectively *p* = 0.468). The sex ratio was also similar between the groups, showing male predominance (1:0.68 and 1:0.68, respectively; *p* = 0.097).

Mean tumor size was significantly different between the PHU group and the PBT group (3.4 cm and 4.3 cm, respectively; *p* < 0.001). The proportion of clinical T stages was significantly different (*p* < 0.001). The PHU group had a higher proportion of cT2 (50.2% in PHU vs. 29.2% in PBT) tumors. The PBT group had a higher proportion of cT3 and cT4 tumors than the PHU group (cT3: 27.3% vs. 12.4% and cT4: 37.1% vs. 28.3%, respectively).

The proportion of tumors was significantly different in terms of classification according to resectability between the PHU and the PBT groups (*p* < 0.001). The PHU group had a higher proportion of resectable and borderline resectable pancreatic cancers (resectable—36.6% vs. 18.2% and borderline resectable: 6.9% vs. 3.1%, respectively) and a lower proportion of metastatic pancreatic cancer (31.0% vs. 54.7%, respectively) than the PBT group. The proportion of locally advanced pancreatic cancers was similar between the groups (25.5% and 24.1% in the PHU and the PBT groups, respectively).

**Figure 1:**
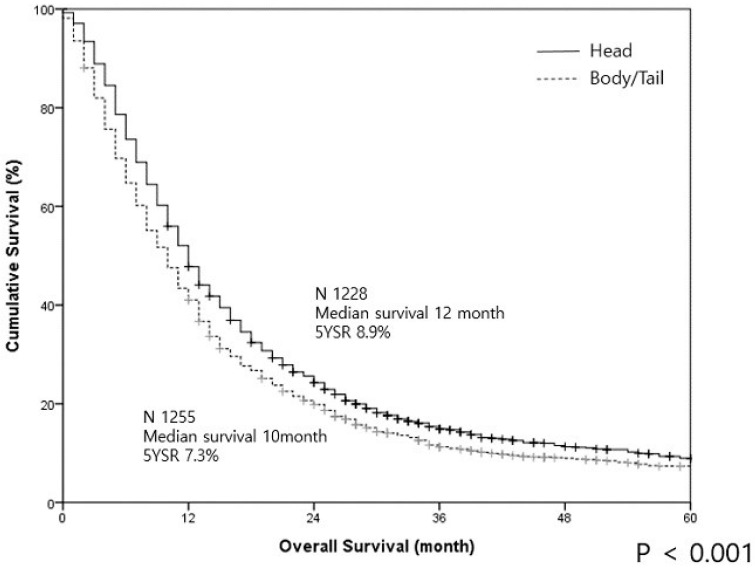
The survival curves of pancreatic cancer in the head/uncinate region and body/tail regions in all patients are illustrated.

The median survival in all patients was 11 months and the 5-year survival rate was 8.1%. The PHU group demonstrated significantly better survival than the PBT group (median survival—12 vs. 10 months, and 5-year survival—8.9% vs. 7.3%, respectively; *p* < 0.001) (Figure 1). 

### 2.2. Demographics of the Patients Who Underwent Resection

Among 705 patients who were advised to undergo curative resection, 28 patients who underwent neoadjuvant treatment and 31 patients who ended up having non-curative surgery were excluded. Thus, 646 patients underwent curative resection. Altogether, 432 (66.9%) patients in the PHU group and 214 (33.1%) patients in the PBT group underwent curative resection. The PHU group had a significantly smaller tumor size, more angiolymphatic invasion and perineural invasion, a lower proportion of T3 and T4 tumors, a higher proportion of N2 and a lower proportion of N0 tumors, greater recurrence, and lower incidence of systemic recurrence, when compared to the PBT group. There were no differences in carcinoembryonic antigen and carbohydrate antigen (CA) 19-9 levels, lymph node (LN) metastasis rate, and the proportion of patients who received adjuvant therapy. Demographics and clinicopathological features are summarized in Table 2.

### 2.3. Survival Analysis of the Patients Who Underwent Resection

**Figure 2:**
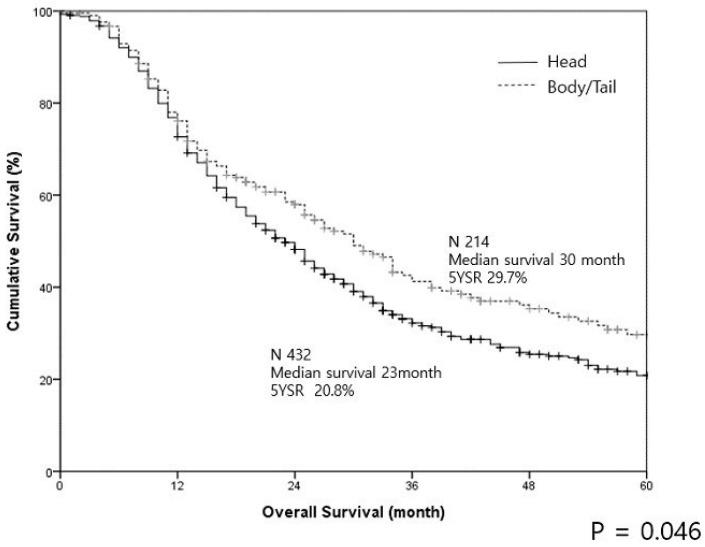
The survival curves of pancreatic cancer in the head/uncinate region and body/tail regions in resected patients are illustrated.

The median survival duration was 25 months and the 5-year survival was 23.6%. For the PHU group, the median survival duration was 23 months and the 5-year survival was 20.8%. For the PBT group, the median survival duration was 30 months and the 5-year survival was 29.7%. Thus, the survival outcome in the PBT group was significantly superior to that in the PHU group (*p* = 0.046) (Figure 2).

**Figure 3:**
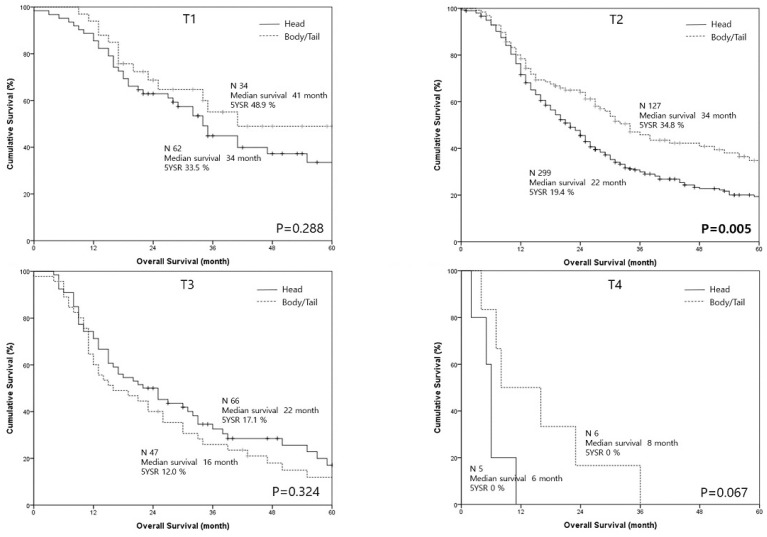
The survival curves of pancreas head/uncinate cancer and pancreas body cancer according to the T categories.

Survival outcomes were compared according to the T category. For T1, T2, and T4 tumors, the PHU group had worse outcomes compared to the PBT group. The difference was not significant for the T1 (median survival 34 vs. 41 months, respectively; *p* = 0.288) and T4 tumors (median survival 6 vs. 8 months, respectively; *p* = 0.067). A significant difference was found in T2 tumors with a median survival of 22 months for the PHU group (5-year survival 19.4%) and a median survival of 34 months for the PHU group (5-year survival 34.8%) (*p* = 0.005, Figure 3). 

In node-negative disease, the PHU group had worse median survival than the PBT group, but the difference was not significant (33 vs. 39 months, respectively; *p* = 0.454). Similarly, in the node-positive disease, the PHU group had worse outcomes than the PBT group, but the difference lacked statistical significance (19 vs. 25 months, respectively; *p* = 0.112).

According to the prognostic groups of the AJCC cancer staging system (edition 8), there were no differences in survival outcomes between the PHU and the PBT groups, in all stages from stage Ia to stage III (Figure S1).

### 2.4. Prognostic Factors of Pancreatic Cancer

Tumor location, histological grade, margin status, angiolymphatic invasion, venous invasion, perineural invasion, T category, N category, adjuvant chemotherapy, adjuvant radiotherapy, and preoperative CA 19-9 were significantly associated with survival. In the multivariate analysis, tumor location did not reach statistical significance (vs. PBT: hazard ratio [HR] 1.174, confidence interval [CI] 0.932–1.478, *p* = 0.173). Histological grade, margin status, angiolymphatic invasion, venous invasion, T4 stage, lymph node metastasis, adjuvant chemotherapy, adjuvant radiotherapy, and preoperative CA 19-9 were independent prognostic factors (Table 3).

When analyzed separately for the PHU and the PBT groups, factors associated with survival in the univariate analysis were similar between the groups and similar to the factors associated with the overall patient population. For the PHU group, all associated categories were similar to those associated with the overall patient population. For the PBT group, T2 stage and preoperative CA 19-9 were not associated with survival, while age was associated with survival, when compared to the overall patient population.

Multivariate analysis showed that poorly differentiated histological grade, angiolymphatic invasion, perineural invasion, T4 stage, N2 stage, adjuvant chemotherapy, adjuvant radiotherapy, and preoperative CA 19-9 were significantly associated with survival in the PHU group. In the PBT group, histological grade, margin status, venous invasion, and adjuvant chemotherapy were significantly associated with survival (Table 4).

## 3. Discussion

The AJCC staging system was revised for the eighth time since its first edition in 2018. Its validity was demonstrated in several studies [5,6,7]. The AJCC staging system always considered pancreatic cancers in terms of the whole pancreas, without dividing the pancreas according to the location, since pancreatic cancers in the head/uncinate process and those in the body/tail share the same prognosis and have comparable tumor biology. However, pancreatic cancer is usually treated according to the location. Many studies investigated pancreatic cancers separately according to the location [2,4,8,9,10,11,12]. Furthermore, many studies investigated whether the subjects underwent distal pancreatectomy or pancreatoduodenectomy, which is a reflection of the location of the tumor [13,14,15,16]. Pancreatic cancer is often not looked at somewhat differently. In this light, it must be clarified whether pancreatic head cancers and pancreatic body or tail cancers have comparable outcomes and oncological behaviors.

Traditionally, pancreatic cancers in the body/tail are believed to have a worse prognosis compared to pancreatic head cancers. This finding was supported by several studies [2,8,9,10,17] and it was also reproduced in the present study. The 5-year survival percentages and the median survival durations were significantly better for the PHU group than for the PBT group, in all pancreatic cancers, regardless of their resectability. The poor outcome of pancreatic cancers in the body/tail is usually explained by their late detection. 

While a pancreatic head cancer might cause obstructive jaundice as the tumor progresses, patients with pancreatic body/tail cancers do not show symptoms until the tumor size increases sufficiently to cause abdominal pain and colon obstruction. In the present cohort, the tumor size measured on the cross-sectional images was significantly greater in the PBT group. Larger tumors reduce the possibility of resectability, which is also reflected in the results of the present study. In the present study, 36.6% of the pancreatic head cancers were deemed resectable, while only 18.2% of the pancreatic body/tail cancers were deemed resectable.

Late detection of the pancreatic body and tail tumors allows them to grow, reducing their resectability. It also increases the possibility of systemic involvement. Other studies that investigated pancreatic cancers according to their locations showed that pancreatic body and tail cancers often present with distant metastases at the time of diagnosis [2,11]. The present study also confirmed a higher proportion of systemic spread at presentation (54.7% in the body/tail cancers and 31.0% in the head/uncinate region cancers).

A completely opposite set of findings was observed when only the resected cases were considered. In the resected cases, pancreatic cancers in the head/uncinate region demonstrated significantly worse survival than those in the body/tail region. Many studies found similar results in resectable pancreatic cancers in the head/uncinate regions [11,12,18], while some studies failed to show worse results for the head region when compared to the body/tail region [9,10,13,14,15,16,19]. However, none of these studies showed significantly worse outcomes in pancreatic body/tail cancers [12].

Studies that demonstrated comparable outcomes between resectable pancreatic cancers in the head and those in the body/tail should be noted for their study population. Studies conducted by Sohn et al. [13], Wade et al. [14], and Brennan et al. [19] published in 2000, 1995, and 1996, respectively, are considered the historic ones. Their study populations were collected from as early as 1984 and up to 1999. During this period, safety and oncological feasibility of pancreatic cancer surgery was more of an issue. Furthermore, adjuvant treatment, which is currently an important part of pancreatic cancer treatment, was not established. The studies from the late 2000s and the 2010s had similar problems regarding patient populations as those associated with the patient populations from the 1980s and the 1990s, even though they included more recent cohorts [9,15,16].

Only one study that included 351 patients showed superior outcomes in the resected pancreatic head cancers, when compared with the resected body/tail cancers [4]. The median survivals of patients with pancreatic head cancers and of those with pancreatic body/tail cancers were 16 and 11 months, respectively. This rather poor survival outcome in patients with resected tumors limited the value of this study. All the other studies reported comparable or superior outcomes in resected pancreatic body/tail cancers than in resected pancreatic head cancers. Therefore, based on the recent literature and the results of the present study, it could be safely concluded that resected pancreatic cancers in the body/tail region have better outcomes than those in the head region. As such is the case, resection of pancreatic body/tail cancers should not be discouraged because of the poor overall prognosis, but rather should be attempted, whenever deemed resectable.

When analyzed according to the T stages, significant difference was observed in survival between the groups for T2 tumors. The PHU group showed worse outcomes than the PBT group for T1 tumors, but the difference was not significant. This finding might perhaps be attributed to small-sized subgroups. Thus, earlier T categories (T1 and T2) demonstrated significantly worse median survival and 5-year survival (24 months and 21.8%) in the PHU group than in the PBT group (34 months and 37.2%) (*p* = 0.003). Meng et al. [12] also found that resected pancreatic head cancers had worse outcomes in the earlier T stages, but significant difference was observed only for the T1 stage.

When stratified according to the N stage and the prognostic groups, the survival rates were not significantly different. There was a tendency toward worse survival for pancreatic head/uncinate cancers in the N0 and LN metastasis groups. For the prognostic groups, pancreatic head/uncinate cancers in stages IB, IIB, and III tended to have worse survival. Re-evaluation using a larger cohort or meta-analysis might clarify the effect of cancer location in each stratified analysis.

There were varying results regarding whether the location of pancreatic cancer was an independent prognostic factor. The present study found that cancer location was not an independent risk factor (head vs. body/tail: HR 1.174, CI 0.932–1.478, *p* = 0.173). Similarly, Ruess et al. [16] and van Erning et al. [10] did not identify location as an independent risk factor. Several other studies suggested that location was a significant risk factor [2,4,9,11,12,18]. Therefore, the status of cancer location as an independent prognostic factor is still controversial and needs further high-level evidence.

The difference in survival outcomes between the locations might be due to plain anatomical differences causing symptoms at different time intervals. There might be additional differences in tumor biology and behavior. To investigate the differences in tumor biology and behavior, clinicopathological features of pancreatic cancers in the head/uncinate process and cancers in the body/tail region were analyzed and compared. Some differences were present, but common risk factors were also observed. Hence, the results are unclear and a definite conclusion cannot be obtained. 

Additionally, differences on genetic and molecular levels should also be considered. The present study did not examine this aspect, but previous studies examined genetic profiles. Birnbaum et al. [20] found differences in 334-gene expression signature between tumors in the head and those in the body/tail. Dreyer et al. [8] reported that tumors might have different molecular pathology, according to their location and the body/tail tumors are enriched with gene programs involved in tumor invasion, epithelial-to-mesenchymal transition, and poor antitumor immune response. This is an important area of research, as the differences on genetic and molecular levels might open a new era of more tailored treatment approaches, according to the location.

The present study had some limitations. The study was retrospective in nature. In addition, the overall patient dataset was acquired through a clinical data warehouse. Hence, more specific variables could not be retrieved in detail. As the present study was performed at a tertiary hospital, many patients visited after being diagnosed at other primary or secondary hospitals, which might have resulted in bias regarding the date of diagnosis. The study population was insufficient for subgroup analyses after stratification.

## 4. Materials and Methods

### 4.1. Study Design

The study was approved by the ethical committee of the Institutional Review Board of Seoul National University Hospital (IRB No. H-1902-012-1006). Seoul National University Hospital’s Clinical Data Warehouse was searched for patients who were diagnosed with pancreatic ductal adenocarcinoma between 2005 and 2016. A retrospective cohort study was performed. 

This research was supported by the Collaborative Genome Program for Fostering New Post-Genome Industry of the National Research Foundation funded by the Ministry of Science and ICT (NRF-2017M3C9A5031591), and by a grant from the Korean Health Technology R and D Project, Ministry of Health and Welfare, Republic of Korea (HI14C2640).

### 4.2. Patient Selection

After the identification of patients with pancreatic cancer from the database, those with multiple tumors in both the head and the body/tail were excluded. Patients who had tumors across the junction of the head and the body were also excluded, as grouping according to the location was ambiguous in these tumors. Data regarding age, sex, tumor location, tumor size on radiological images, clinical feature (T) classification, and classification based on resectability were collected. 

Further subgroup analysis was performed for patients who underwent resection with curative intent. Among all patients, 646 patients who underwent resection of pancreatic cancer with curative intent were examined. Patients who underwent only palliative operation including bypass or open biopsy were excluded. Since neoadjuvant treatment can alter the final pathological staging, patients who received neoadjuvant therapy were also excluded. Detailed information about the demographic and clinicopathological factors of these patients was obtained through a thorough review of their electronic medical records.

### 4.3. Determination of Tumor Location and Clinical T Staging

Computed tomography (CT) or magnetic resonance imaging (MRI) records of all patients were reviewed. An imaginary tangential line over the left border of the superior mesenteric vein or the portal vein was drawn on the CT image. The head/uncinate pancreatic cancer group (PHU) was defined as patients with tumors on the right side of this line. The body/tail pancreatic cancer group (PBT) was defined as patients with tumors on the left side of this line.

Clinical T staging was performed according to the AJCC staging system (edition 8) for pancreatic cancer. Tumor size was measured using CT and MRI.

### 4.4. Definition of Survival and Data Collection 

Overall survival was used for the analysis. It was defined as the interval between the date of diagnosis and the date of death or the last follow-up. Survival status was acquired from the Ministry of Interior and Safety of Korea. Patients who were alive on 20 February 2019 were censored.

### 4.5. Statistical Analysis

Fisher’s exact test and chi-squared test were used to compare categorical variables and unpaired two-sided Student’s t-test was used to compare continuous variables between patients with tumors located in the head/uncinate process and patients with tumors in the body/tail. The Kaplan-Meier method with log-rank test was used for survival analysis. Cox regression test was used for the univariate and the multivariate analyses. A *p*-value < 0.050 was considered to be statistically significant. IBM SPSS statistics for Windows version 24 (IBM Corp., Armonk, NY, USA) was used for statistical analyses.

## 5. Conclusions

The prognosis of pancreatic cancers differed according to the location of the tumors. Pancreatic head cancers showed a better overall prognosis than pancreatic body/tail cancers, which might be related to a higher proportion of systemic involvement in the latter. On the contrary, resected pancreatic head cancers showed a worse prognosis than resected pancreatic body/tail cancers, especially in the earlier T stages. Tumor location was not an independent risk factor for pancreatic cancer.

## Figures and Tables

**Table 1:** Demographics and clinicopathological features of overall patients.

Variables	Total (*N* = 2483)	PHU (*N* = 1228)	PBT (*N* = 1255)	*p*-Value
Age (years)	64.1 (23–94)	64.3 (28–94)	64.0 (23–93)	0.468
Sex (Male:Female)	1:0.68	1:0.68	1:0.68	0.970
Tumor size (cm)	3.8 (0.1–15.5)	3.4 (0.1–8.5)	4.3 (0.1–15.5)	<0.001
Clinical T stage				<0.001
T1	192 (7.7%)	113 (9.2%)	79 (6.3%)	
T2	983 (39.6%)	616 (50.2%)	367 (29.2%)	
T3	495 (19.9%	152 (12.4%)	343 (27.3%)	
T4	813 (32.7%)	347 (28.3%)	466 (37.1%)	
Resectability				<0.001
Resectable	677 (27.3%)	449 (36.6%)	228 (18.2%)	
Borderline	124 (5.0%)	85 (6.9%)	39 (3.1%)	
Locally advanced	615 (24.8%)	313 (25.5%)	302 (24.1%)	
Distant metastasis	1067 (43.0%)	381 (31.0%)	686 (54.7%)	
Operation				<0.001
Non-resectable	1778 (71.6%)	766 (62.4%)	1012 (80.6%)	
Preemptive-resectable	705 (28.4%)	462 (37.6%)	243 (19.4%)	

PHU—tumors in the pancreas head or uncinated process; PBT—tumors in the pancreas body and tail. Continuous variables were expressed as median (range). Statistical significance when *p* value < 0.05.

**Table 2:** Demographics and clinicopathological features of resected patients.

Variables	Total	PHU	PBT	*p*-Value
	(*N* = 646)	(*N* = 432)	(*N* = 214)	
Age (years)	64.6 (29–89)	63.7 (29–88)	66.4 (35–89)	0.001
Sex (Male:Female)	1:0.7	1:0.67	1:0.75	0.552
Preoperative CEA (ng/mL)	4.1 (0.5–179.1)	3.5 (0.5–63)	5.2 (0.5–179.1)	0.124
Preoperative CA19-9 (U/mL)	1040.7 (0.1–37800)	1109.2 (0.1–28700)	901.7 (1–37800)	0.421
Operation name				<0.001
PPPD	251 (38.9%)	251 (58.1%)	0	
Whipple’s operation	163 (25.2%)	163 (37.7%)	0	
Distal pancreatectomy	190 (29.4%)	0	190 (88.8%)	
Subtotal pancreatectomy	17 (2.6%)	0	17 (7.9%)	
Total pancreatectomy	23 (3.6%)	17 (3.9%)	23 (3.6%)	
Central pancreatectomy	2 (0.3%)	1 (0.2%)	2 (0.3%)	
Complication	264 (40.9%)	199 (46.1%)	65 (30.4%)	<0.001
Adjuvant therapy				
Chemotherapy	516 (79.9%)	344 (79.6%)	172 (80.4%)	0.917
Radiotherapy	343 (53.1%)	231 (53.5%)	112 (52.3%)	0.802
Tumor size(cm)	3.3 (0.2–12.2)	3.1 (0.2–8.0)	3.5 (0.5–12.2)	0.008
Differentiation				0.053
Well Differentiated	43 (7.1%)	23 (5.6%)	20 (10.2%)	
Moderate Differentiated	488 (80.7%)	340 (83.1%)	148 (75.5%)	
Poorly Differentiated	74 (12.2%)	46 (11.2%)	28 (14.3%)	
Resection margin status				0.198
Tumor free	549 (85.0%)	373 (86.3%)	176 (82.2%)	
Presence of tumor	97 (15.0%)	59 (13.7%)	38 (17.8%)	
Angiolymphatic invasion				<0.001
Negative	354 (55.0%)	213 (49.4%)	141 (66.2%)	
Positive	290 (45.0%)	218 (50.6%)	72 (33.8%)	
Vascular invasion				0.200
Negative	389 (60.3%)	252 (58.5%)	137 (64.0%)	
Positive	256 (39.7%)	179 (41.5%)	77 (36.0%)	
Perineural invasion				0.014
Negative	108 (16.7%)	61 (14.1%)	47 (22.0%)	
Positive	538 (83.3%)	371 (85.9%)	167 (78.0%)	
T stage				0.041
T1	96 (14.9%)	62 (14.4%)	34 (15.9%)	
T2	426 (65.9%)	299 (69.2%)	127 (59.3%)	
T3	113 (17.5%)	66 (15.3%)	47 (22.0%)	
T4	11 (1.7%)	5 (1.2%)	6 (2.8%)	
N stage				0.033
N0	252 (39.0%)	160 (37.0%)	92 (43.0%)	
N1	270 (41.8%)	177 (41.0%)	93 (43.5%)	
N2	124 (19.2%)	95 (22.0%)	29 (13.6%)	
Stage				0.171
Ia	65 (10.1%)	38 (8.8%)	27 (12.6%)	
Ib	150 (23.2%)	101 (23.4%)	49 (22.9%)	
IIa	34 (5.3%)	20 (4.6%)	14 (6.5%)	
IIb	265 (41.0%)	175 (40.5%)	90 (42.1%)	
III	132 (20.4%)	98 (22.7%)	34 (15.9%)	
Recurrence				0.006
No	211 (32.7%)	125 (29.0%)	86 (40.2%)	
Yes	434 (67.3%)	306 (71.0%)	128 (59.8%)	
Recurrence type				0.028
Local	66 (15.2%)	54 (17.7%)	12 (9.4%)	
Systemic	367 (84.8%)	251 (82.3%)	116 (90.6%)	

PHU—tumors in the pancreas head or uncinated process; PBT—tumors in the pancreas body and tail; PPPD—pylorus-preserving pancreatoduodenectomy. Continuous variables were expressed as median (range). Statistically significant when *p* value < 0.05.

**Table 3:** Univariate and multivariate analysis comparing the 5-year survival rates in resected patients.

Variables	Univariate			Multivariate		
	*n*	5 YSR, %	*p* Value	HR	95%CI	*p* Value
Sex						
Male	380	21.2				
Female	266	27.3	0.269			
Age(years)						
<65	301	24.0				
≥65	345	23.6	0.070	1.095	0.881–1.362	0.414
Site of tumor						
Head	432	20.8				
Body, tail	214	29.7	0.046	1.174	0.932–1.478	0.173
Complication						
No	382	22.7				
Yes	264	24.9	0.986			
Histologic grade			<0.001			<0.001
Well differentiated	43	45.0				
Moderate differentiated	488	21.9	<0.001	2.092	1.306–3.351	0.002
Poorly differentiated	74	11.2	<0.001	3.133	1.834–5.354	<0.001
Margin						
Negative	549	26.5				
Positive	97	5.3	<0.001	1.471	1.126–1.923	0.005
Angiolymphatic invasion						
Negative	354	31.0				
Positive	290	13.7	<0.001	1.473	1.191-1.823	<0.001
Venous invasion						
Negative	389	30.1				
Positive	256	11.9	<0.001	1.309	1.059–1.618	0.013
Perineural invasion						
Negative	108	42.3				
Positive	538	19.7	<0.001	1.250	0.895–1.746	0.191
T stage			<0.001			0.008
T1	96	37.9				
T2	426	23.7	0.001	1.143	0.814–1.604	0.440
T3	113	15.0	<0.001	1.278	0.858–1.902	0.227
T4	11	0.0	<0.001	4.874	2.228–10.664	<0.001
N stage			<0.001			0.005
N0	252	37.3				
N1	269	17.0	<0.001	1.271	1.002–1.613	0.048
N2	125	8.2	<0.001	1.611	1.207–2.150	0.001
Adjuvant Chemotherapy						
Yes	516	25.0				
No	130	18.4	<0.001	1.626	1.206–2.193	0.001
Adjuvant Radiotherapy						
Yes	343	27.3				
No	303	20.9	<0.001	1.417	1.103–1.821	0.006
Preoperative CEA						
<5.0 ng/mL	524	24.8				
≥5.0 ng/mL	94	21.9	0.061	1.168	0.884–1.544	0.274
Preoperative CA19-9						
<37.0 U/mL	187	38.9				
≥37.0 U/mL	446	18.1	<0.001	1.600	1.258–2.037	<0.001

YSR—year survival rate; HR—hazard ratio; CI—confidence interval. The variables with *p*-value less than 0.1 in univariate analysis were included in the multivariate analysis.

**Table 4:** Comparison of independent risk factors of pancreatic cancer in PHU and PBT.

Variables	PHU						PBT					
	Univariate			Multivariate			Univariate			Multivariate		
	*n*	5YSR, %	*p* Value	HR	95%CI	*p* Value	*n*	5YSR, %	*p* Value	HR	95%CI	*p* Value
Sex												
Male	258	16.6					122	31.2				
Female	174	26.9	0.254				92	27.0	0.968			
Age (years)												
<65	220	19.3					81	39.0				
≥65	212	23.5	0.272				133	24.3	0.020	1.135	0.740–1.740	0.561
Complication												
No	233	18.8					149	28.7				
Yes	199	22.8	0.645				65	33.1	0.883			
Histologic grade			<0.001			0.001			0.002			0.004
Well differentiated	23	27.7					20	69.1				
Moderate differentiated	340	19.7	0.035	1.368	0.781–2.396	0.273	148	27.5	0.003	3.632	1.503–8.777	0.004
Poorly differentiated	46	12.3	<0.001	2.692	1.419–5.108	0.002	28	9.0	<0.001	3.609	1.318–9.885	0.013
Margin												
Negative	373	22.5					176	35.8				
Positive	59	5.8	0.006	1.165	0.823–1.649	0.388	38	4.4	<0.001	2.431	1.580–3.743	<0.001
Angiolymphatic invasion												
Negative	213	29.0					141	34.4				
Positive	218	12.4	<0.001	1.393	1.079–1.799	0.011	72	18.5	0.002	1.412	0.952–2.095	0.087
Venous invasion												
Negative	252	26.5					137	37.8				
Positive	179	11.1	<0.001	1.239	0.966–1.590	0.092	77	14.3	<0.001	1.523	1.014–2.288	0.042
Perineural invasion												
Negative	61	40.7					47	45.2				
Positive	371	17.4	<0.001	1.581	1.033–2.420	0.035	167	25.1	0.036	0.669	0.389–1.149	0.145
T stage			<0.001			0.004			<0.001			0.034
T1	62	33.5					34	48.9				
T2	299	19.4	0.004	1.303	0.868–1.956	0.201	127	34.8	0.158	0.827	0.445–1.534	0.546
T3	66	17.1	0.034	1.215	0.734–2.012	0.448	47	12.0	0.001	1.433	0.749–2.742	0.278
T4	5	0.0	<0.001	13.539	4.519–40.565	<0.001	6	0.0	<0.001	2.336	0.769–7.096	0.134
N stage			<0.001			0.037			0.004			0.180
N0	160	33.2					92	45.3				
N1	177	16.4	<0.001	1.223	0.920–1.627	0.166	92	18.4	0.005	1.474	0.936–2.319	0.094
N2	95	7.3	<0.001	1.571	1.114–2.214	0.010	30	12.2	0.006	1.554	0.867–2.784	0.139
Adjuvant Chemotherapy												
Yes	344	22.5					172	30.6				
No	88	14.1	<0.001	1.511	1.074–2.125	0.018	42	26.2	0.034	2.466	1.463–4.156	0.001
Adjuvant Radiotherapy												
Yes	231	25.1					112	32.1				
No	201	17.0	<0.001	1.482	1.108–1.983	0.008	102	29.2	0.080	1.279	0.793–2.064	0.313
Preoperative CEA												
<5.0 ng/mL	354	22.1					170	30.9				
≥5.0 ng/mL	60	18.5	0.053	1.277	0.917–1.778	0.148	34	27.3	0.459			
Preoperative CA19-9												
<37.0 U/mL	177	39.9					70	35.6				
≥37.0 U/mL	307	14.2	<0.001	1.621	1.199–2.192	0.002	139	26.8	0.120			

PHU—tumors in the pancreas head or uncinated process; PBT—tumors in the pancreas body and tail; YSR—year survival rate; HR—hazard ratio; CI—confidence interval; The variables which *p*-value was less than 0.1 in univariate analysis were included in the multivariate analysis.

## Supplementary Materials

The following are available online at https://www.mdpi.com/2072-6694/12/8/2036/s1. Figure S1: The survival curves of pancreas head/uncinate cancer and pancreas body cancer in terms of the prognostic group, according to the eighth edition of AJCC cancer staging system.
