# Supplementary material for: The Role of Location of Tumor in the Prognosis of the Pancreatic Cancer
Source: Cancers (Basel). 2020 Jul 24;12(8):2036. doi: 10.3390/cancers12082036 (PMC7465041; doi:10.3390/cancers12082036)
Supplement: Supplementary file 1 [file cancers-12-02036-s001.pdf]

Supplementary Materials:

# The Role of Location of Tumor in the Prognosis of the Pancreatic Cancer


**Mirang Lee, Wooil Kwon, Hongbeom Kim, Yoonhyeong Byun, Youngmin Han, Jae Seung Kang, Yoo Jin Choi and Jin-Young Jang**


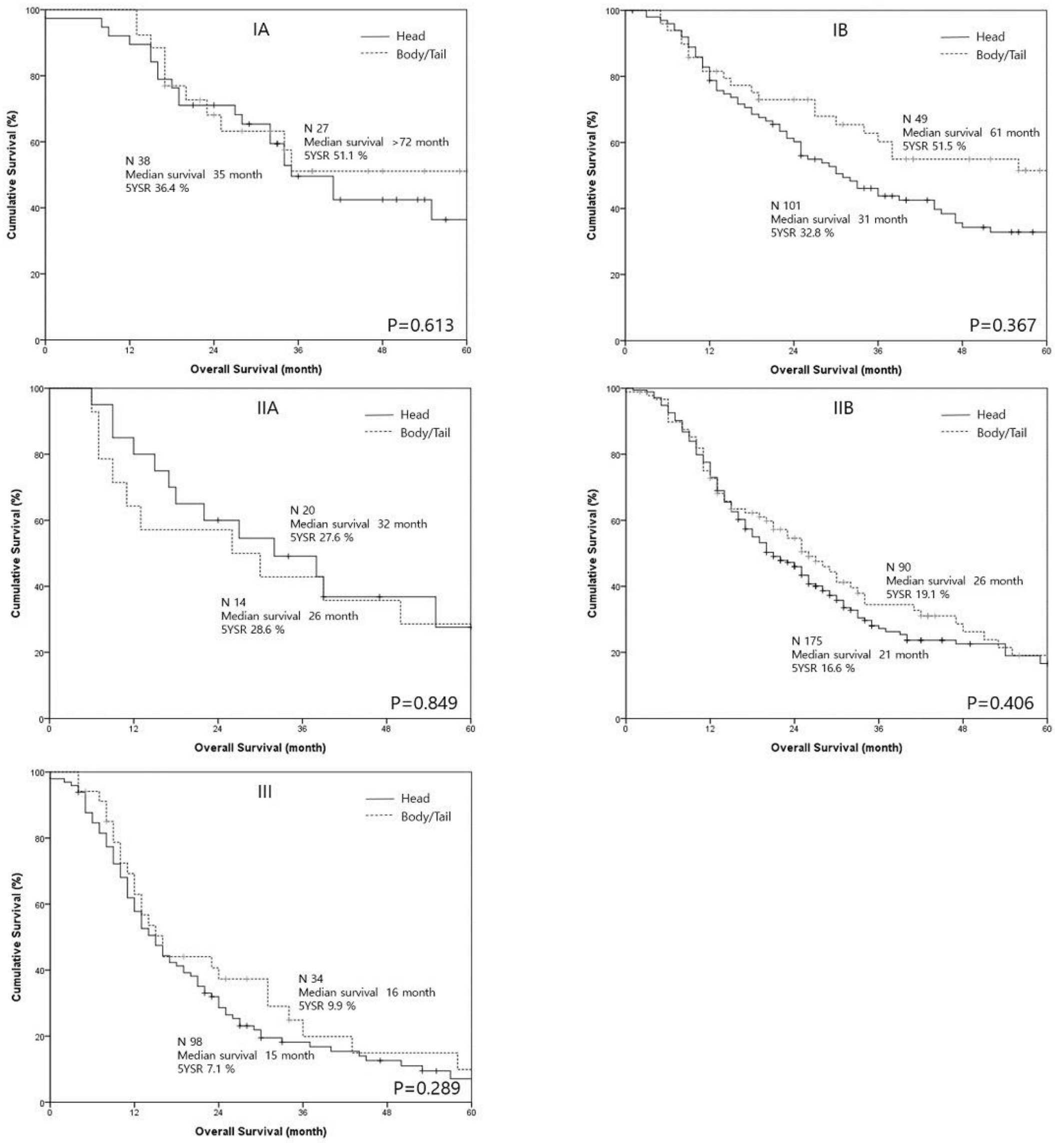


**Figure S1.** The survival curves of pancreas head/uncinate cancer and pancreas body cancer according to prognostic group according to the eighth edition of AJCC cancer staging system.
